# Growth factor and macromolecular crowding supplementation in human tenocyte culture^☆^

**DOI:** 10.1016/j.bbiosy.2021.100009

**Published:** 2021-01-30

**Authors:** Dimitrios Tsiapalis, Stephen Kearns, Jack L. Kelly, Dimitrios I. Zeugolis

**Affiliations:** a Regenerative, Modular & Developmental Engineering Laboratory (REMODEL), Biomedical Sciences Building, National University of Ireland Galway (NUI Galway), Galway, Ireland; b Science Foundation Ireland (SFI) Centre for Research in Medical Devices (CÚRAM), Biomedical Sciences Building, National University of Ireland Galway (NUI Galway), Galway, Ireland; c Merlin Park University Hospital, Galway, Ireland; d Galway University Hospital, Galway, Ireland; e Regenerative, Modular & Developmental Engineering Laboratory (REMODEL), Faculty of Biomedical Sciences, Università della Svizzera Italiana (USI), Lugano, Switzerland

**Keywords:** Tendon tissue engineering, *In vitro* microenvironment, Tenogenic phenotype, Media supplements, Excluded volume effect

## Abstract

Cell-assembled tissue engineering strategies hold great potential in regenerative medicine, as three-dimensional tissue-like modules can be produced, even from a patient's own cells. However, the development of such implantable devices requires prolonged *in vitro* culture time, which is associated with cell phenotypic drift. Considering that the cells *in vivo* are subjected to numerous stimuli, multifactorial approaches are continuously gaining pace towards controlling cell fate during *in vitro* expansion. Herein, we assessed the synergistic effect of simultaneous and serial growth factor supplementation (insulin growth factor-1, platelet-derived growth factor *ββ,* growth differentiation factor 5 and transforming growth factor *β*3) to macromolecular crowding (carrageenan) in human tenocyte function; collagen synthesis and deposition; and gene expression. TGF*β*3 supplementation (without/with carrageenan) induced the highest (among all groups) DNA content. In all cases, tenocyte proliferation was significantly increased as a function of time in culture, whilst metabolic activity was not affected. Carrageenan supplementation induced significantly higher collagen deposition than groups without carrageenan (without/with any growth factor). Of all the growth factors used, TGF*β*3 induced the highest collagen deposition when used together with carrageenan in both simultaneous and serial fashion. At day 13, gene expression analysis revealed that TGF*β*3 in serial supplementation to carrageenan upregulated the most and downregulated the least collagen- and tendon- related genes and upregulated the least and downregulated the most osteo-, chondro-, fibrosis- and adipose- related trans-differentiation genes. Collectively, these data clearly advocate the beneficial effects of multifactorial approaches (in this case, growth factor and macromolecular crowding supplementation) in the development of functional cell-assembled tissue surrogates.

## Highlights

• MMC induced significantly higher collagen deposition than groups without MMC (without or with any growth factor).
• TGF*β3* induced the highest collagen deposition when used together with MMC.
• Gene expression analysis revealed the beneficial effect of TGF*β*3 in serial supplementation to MMC in tenocyte cultures.
• These data advocate the beneficial effects of multifactorial tissue engineering approaches.

## Graphical abstract

**Figure:**
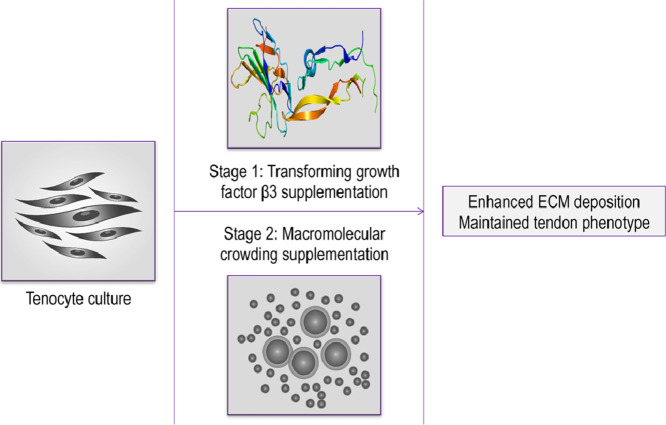

## 1 Introduction

In the context of tissue engineering and regenerative medicine, scaffold-based approaches are frequently associated with foreign body immunological reactions [1] and impaired vascularization [2]. Thus, particular emphasis has been given to scaffold-free/cell-sheet tissue engineering strategies to restore injured and damaged tissues and organs [3,4], as they capitalise the cells’ inherent ability to synthesise tissue-specific extracellular matrix (ECM). This bottom-up paradigm enables the formation of sophisticated tissue-like surrogates with low immunological response and high levels of biomimicry, vascularisation capacity and regenerative potential, as the delivered cells and their rich in bioactive molecules/tropic factors secretome are now entangled within their own intertwined network of deposited ECM [5], [6], [7], [8]. Yet again, there are limitations regarding this cell-based self-assembly approach, such as the high cell seeding density required, which brings into consideration the issue of cell sourcing [9], and the prolonged culture time needed to create an implantable construct (e.g. for 35 days for cornea [10], 42 days for vascular [11]) which brings into consideration the issue of cell phenotype maintenance during prolonged *ex vivo* culture [12]. Indeed, passaged primary cells experience epigenetic changes leading to an altered protein and gene profile, phenotypic drift, loss of function and senescence, ultimately reducing the efficacy and efficiency of cell-based therapies [13], [14], [15], [16], [17], [18]. Hence, it becomes imminent to develop biomimetic cell expansion methods that will accelerate the development of tissue-like modules *in vitro*, whilst maintaining cellular phenotype and function.

Although tendon derived cells are preferred for tendon tissue engineering [19,20], serial passaging is associated with phenotypic drift [21]. To this end, various biophysical (e.g. surface topography [22, 23], mechanical stimulation [24]), biochemical (e.g. physiological low oxygen tension [25], media supplements [26]), biological (e.g. co-culture [27], growth factors (GFs) [28]), alone or in combination [29], [30], [31], [32], microenvironmental cues mimicking the native tendon milieu are employed as a means to either maintain tendon derived cell phenotype or to differentiate other cell types towards tenogenic lineage, albeit with variable degree of efficiency. among the different *in vitro* microenvironment modulators, the use of GFs has been extensively advocated due to their potent and multifunctional value in tendon regeneration and increased tendon-derived cell proliferation, expression of tenogenic markers and production of tendon-specific ECM [33], [34], [35], [36], [37], [38]. In particular, 100 ng/ml insulin growth factor 1 (IGF1) [39,40], 50 ng/ml of platelet-derived growth factor *ββ* (PDGF*ββ*) [31,41], 100 ng/ml growth differentiation factor 5 (GDF5) [31,42] and 20 ng/ml transforming growth factor *β*3 (TGF*β*3) [43], [44], [45] have been shown not only to enhance tenocyte proliferation, ECM synthesis and prolonged expression of tenogenic markers, but also to induce tenogenic differentiation of stem cells (both to cells from various species).

However, GF supplementation alone is not sufficient to substantially enhance ECM deposition, as in the conventional dilute culture systems, the enzymatic procollagen processing to stable collagen is very slow [46,47]. Macromolecular crowding (MMC) has been shown to accelerate biochemical reactions and biological activities by several orders of magnitude, following the principles of excluded volume effect [48,49]. In eukaryotic cell culture, the addition of macromolecules in the culture media prohibits the diffusion of procollagen and *N*- and *C*- proteinases, resulting in enhanced and accelerated ECM deposition (within 48 h over 80-fold increase) [50], [51], [52], [53]. Macromolecular crowding, alone [54], [55], [56], [57] or in combination with physiological oxygen tension [58], [59], [60], [61] or mechanical stimulation [62], has already shown promising results in controlling permanently differentiated and stem cell fate *in vitro*. Considering that GFs are potent cell fate modulators and MMC is key inducer of enhanced and accelerated ECM deposition, herein, we ventured to assess the synergistic effect of IGF1, PDGF*ββ*, GDF5 or TGF*β*3 and MMC in human tenocyte culture. The driving hypothesis is that GF supplementation will maintain tenogenic phenotype and induce tendon-specific ECM synthesis, whilst MMC will accelerate the deposition of the tendon-like *de novo* synthesised ECM.

## 2 Material and methods

### 2.1. Human tenocyte isolation and culture

Human tendons were obtained from patients without tendinopathy undergoing tendon surgeries at Galway University Hospital, Galway, Ireland and at Bon Secours Hospital, Galway, Ireland. Appropriate licences, ethical approvals and informed consent forms were in place. Tenocytes were extracted using the migration method [63]. Briefly, tendons were cleaned aseptically with a scalpel to remove all paratendon, fat and muscle tissues. Subsequently, tendons were cut into small pieces and placed into 6-well plates. Tendon segments were supplemented with 5 ml of culture media containing Dulbecco's modified Eagle medium (DMEM, Sigma Aldrich, Ireland), 10% foetal bovine serum (FBS, Sigma Aldrich, Ireland) and 1% penicillin-streptomycin (Sigma Aldrich, Ireland) and placed at 37 °C in a humidified atmosphere of 5% CO_2_. Culture medium was changed every three days. After a few days, the first colonies of tenocytes were noticed around the tendon sections. When the migrated tenocytes reached 80–90% confluency, they were treated with trypsin/ethylenediaminetetraacitic acid (EDTA) solution (Sigma Aldrich, Ireland) and sub-cultured in T-175 tissue culture flasks (Sarstedt, Germany).

### 2.2 GF and MMC supplementation

At passage three, tenocytes were seeded at 25,000 cells/cm^2^ in 24-well plates and allowed to attach for 24 h. The culture media was removed and replaced with culture media containing 100 *μ*M L-ascorbic acid phosphate (Sigma Aldrich, Ireland) to induce collagen synthesis and 100 ng/ml recombinant human IGF1 (R&D Systems, UK), 50 ng/ml recombinant human PDGF*ββ* (PeproTech EC, UK), 100 ng/ml recombinant human GDF5 (R&D Systems, UK) or 20 ng/ml recombinant human TGF*β*3 (PeproTech EC, UK) with and without 50 *μ*g/ml of carrageenan (CR, mixture of κ and lesser amounts of λ CR, C1013, Sigma Aldrich, Ireland). The CR was added either simultaneously with or in serial fashion to each GF as described below. The rational of this approach was to decipher the potential effect of CR to the GF activity, as CR has been shown to act as selective GF antagonist. Samples without GF and without/with CR were used as controls. Culture media was changed every 3 days. Analysis was performed after 4, 7, 10 and 13 days. Simultaneous administration of GFs and CR: Day 0: cell seeding; Day 1: GF and CR treatment; Day 4: 1st timepoint and GF/CR treatment; Day 7: 2nd timepoint and GF/CR treatment; Day 10: 3rd timepoint and GF/CR treatment; Day 13: 4th timepoint. Serial administration of GFs and CR: Day 0: cell seeding; Day 1: GF treatment; Day 2: CR administration; Day 4: 1st timepoint and GF treatment; Day 5: CR administration; Day 7: 2nd timepoint and GF treatment; Day 8: CR administration; Day 10: 3rd timepoint and GF treatment; Day 11: CR administration; Day 13: 4th timepoint.

### 2.3 DNA quantification

DNA quantification was performed using Quant-iT™ PicoGreen^Ⓡ^ dSDNA assay kit (Invitrogen, Ireland) to evaluate the effect of GF and CR supplementation on tenocyte proliferation at each timepoint according to the manufacturer's guidelines. Double-stranded DNA (dsDNA) was extracted using three freeze-thaw cycles after adding 250 *μ*l of nucleic acid free water per well. Afterwards, 100 *μ*l of each sample transferred into 96-well plate. A standard curve was generated using 0, 5, 10, 25, 50, 100, 500 and 1000 ng/ml DNA concentrations. 100 *μ*l of 1:200 dilution of Quant-iT™ PicoGreen^Ⓡ^ reagent was then added to each well and the plate was read using a micro-plate reader (Varioskan Flash, Thermo Scientific, Ireland) with an excitation wavelength of 480 nm and an emission wavelength of 525 nm.

### 2.4 Cell metabolic activity assessment

alamarBlue^Ⓡ^ assay (Invitrogen, USA) was carried out to assess the effect of GF and CR supplementation on the tenocytes’ metabolic activity at each timepoint, as per manufacturer's protocol. Briefly, at the end of each culture timepoint, tenocytes were washed with Hanks’ Balanced Salt solution (HBSS, Sigma Aldrich, Ireland) and then alamarBlue^Ⓡ^ solution (10% alamarBlue^Ⓡ^ in HBSS) was added. After 3 h of incubation at 37 °C and 5% CO_2_, absorbance was measured at 550 nm and 595 nm using Varioskan Flash spectral scanning multimode reader (Thermo Scientific, UK). Cell metabolic activity was expressed in terms of% reduction of the alamarBlue^Ⓡ^ dye and normalized to the control group without GF and without CR.

2.5. Cell nuclei counting

Nuclei were counterstained with 4′,6-diamidino-2-phenylindole (DAPI, Invitrogen, USA; see Section 2.7.). Fluorescent images were captured with an Olympus IX-81 inverted fluorescence microscope and images were further processed with ImageJ software. Nuclei were counted to obtain cell number per area at the different timepoints.

### 2.6 Collagen extraction and sodium dodecyl sulphate-polyacrylamide gel electrophoresis (SDS-PAGE) analysis

At each timepoint collagen extraction from the cell layers and SDS-PAGE was performed [64]. Briefly, at days 4, 7, 10 and 13 cell layers were digested with porcine gastric mucosa pepsin (Sigma Aldrich, Ireland) for 2 h at 37 °C with continuous shaking and subsequent neutralisation with 1 N NaOH. The samples were appropriately diluted with distilled water and 5 x sample buffer and heated at 95 °C for 5 min. 10 *μ*l per sample solution per well was loaded on the gel (5% running gel/3% stacking gel). 0.25 mg/ml bovine skin collagen type I (Symatese Biomateriaux, France) was used as reference standard. Electrophoresis was performed in a Mini-PROTEAN Tetra Electrophoresis System (Bio-Rad, Ireland) by applying potential difference of 50 mV for the initial ~30 min and then 110 mV for the remaining time (~ 60 min). The gels were stained using the SilverQuest™ (Invitrogen, Ireland) silver stain kit, according to the manufacturer's protocol. Images of the gels were taken after brief washing with water. To quantify the cell-produced collagen type I deposition, the relative densities of collagen *α*1(I) and *α*2(I) chains were evaluated using ImageJ software (NIH, USA) and correlated to the *α*1(I) and *α*2(I) chain bands densities of the reference standard collagen type I.

### 2.7 Immunocytochemistry analysis

For immunocytochemistry analysis, tenocytes were seeded in 48 well plates at 25,000 cells/cm^2^ and were cultured as described in Section 2.2. At each timepoint (4, 7, 10 and 13 days), culture media was removed, and the cell layers were washed with HBSS and fixed with 2% paraformaldehyde (PFA, Sigma Aldrich, Ireland) at ambient temperature for 30 min. After three washes with phosphate buffered saline (PBS, Sigma Aldrich, Ireland), cells were incubated with 3% bovine serum albumin (BSA) in PBS for 30 min to block nonspecific binding. Samples were then incubated overnight at 4 °C with primary antibodies for collagen type I (mouse monoclonal, Abcam, Ireland) or collagen type III (rabbit polyclonal, Abcam, Ireland) diluted 1:200 in 3% BSA in PBS. After three washes in PBS, bound antibodies were visualised using AlexaFluor^Ⓡ^ 488 donkey anti-mouse (Invitrogen, USA) or AlexaFluor^Ⓡ^ 488 goat anti-rabbit (Invitrogen, USA) diluted 1:400 in PBS for 1 h. The cell nuclei were stained with DAPI (Invitrogen, USA). Images were taken with an Olympus IX-81 inverted fluorescence microscope (Olympus Corporation, Japan) and fluorescent area per image was quantified using ImageJ software (NIH, USA) and normalised per cell number.

### 2.8 Gene analysis

At each timepoint (4, 7, 10 and 13 days) gene analysis (**Supplementary Table S1** provides the list of genes and the sequence of their primers) was conducted using a RealTime ready Custom Panel (Roche, Germany) to investigate the effect of GF and CR supplementation on the expression of the collagenous genes [prolyl 4-hydroxylase subunit alpha 1 (P4HA1), prolyl 4-hydroxylase subunit alpha 2 (P4HA2), procollagen-lysine,2-oxoglutarate 5-dioxygenase 1 (PLOD1), procollagen-lysine,2-oxoglutarate 5-dioxygenase 2 (PLOD2), collagen type I alpha 1 (COL1A1), collagen type III alpha 1 (COL3A1)]; tenogenic markers [scleraxis (SCXA), tenomodulin (TNMD), tenascin C (TNC), Mohawk (MKX), biglycan (BGN), elastin (ELN), fibromodulin (FMOD)]; osteogenic markers [runt related transcription factor 2 (RUNX2), bone gamma carboxyglutamate protein – osteocalcin (BGLAP), integrin binding sialoprotein (IBSP)]; chondrogenic markers [collagen type II alpha 1 (COL2A1), aggrecan (ACAN), SOX9]; fibrosis marker [heat shock protein 47 (SERPINH1)]; and adipogenic marker [fatty acid binding protein 4 (FABP4)]. Total RNA was isolated using TRI Reagent^Ⓡ^ (Sigma Aldrich, Ireland) for 5 min at ambient temperature to lyse the cells. Afterwards, the TRI Reagent^Ⓡ^ was collected, chloroform was added and was vortexed for 15 s and incubated at ambient temperature for 5 min. The solution was centrifuged and the upper aqueous phase containing the RNA was collected and mixed with 70% ethanol. The solution was then purified using the High Pure isolation kit (Roche, Germany). RNA concentration and quality were analysed using the NanoDrop 1000 (Thermo Scientific, UK) and the Agilent 2100 Bioanalyser (Agilent Technologies, Ireland). RNA was transcribed to cDNA using the Transcriptor First Strand cDNA synthesis kit (Roche, Germany) and 1 *μ*g of RNA sample was used in all the groups. After cDNA synthesis, 1 *μ*l of cDNA was added to 9 *μ*l of Probes Master into a RealTime ready custom 384 well plate (Roche, Germany). Negative controls of empty wells and non-transcribed RNA were added in the study and the plate was run in the LightCycler^Ⓡ^ 480 Instrument (Roche, Germany). Genes were normalised to the housekeeper gene Ribosomal Protein Lateral Stalk Subunit P0 (RPLP0) and fold change was obtained using the 2-ΔΔCΤ. Z-scores of fold change were calculated and relevant up- or down- regulations were accepted when the score was at least three standard deviations away from the mean value of fold change for each gene.

### 2.9 Statistical analysis

Unless otherwise specified, all experiments were conducted in triplicates and data are expressed as mean ± standard deviation. MINITAB (version 17; Minitab, Inc.) was used for statistical analysis. Normality test (Anderson-Darling normality test) and equality of variances tests (Bonett's test and Levene's test) were conducted. Parametric analysis was performed using one-way analysis of variance (Tukey's test) for multiple comparisons or 2 sample *t*-test for pair wise comparisons. Non-parametric analysis was performed using Kruskal-Wallis test for multiple comparisons when the assumptions of parametric analysis were violated. Statistical significance was accepted at *p* < 0.05.

## 3 Results

### 3.1 DNA, metabolic activity and proliferation analyses

IGF1 (at days 10 and 13) and TGF*β*3 (at all timepoints) supplementation (without/with CR) significantly (*p* < 0.05) increased DNA content (**Supplementary Figure S1**). With respect to PDGF*ββ* (at all timepoints) and GDF5 (at days 7, 10 and 13) supplementation, groups without CR and in serial fashion to CR induced the highest (*p* < 0.05) DNA content (**Supplementary Figure S1**). No significant differences (*p* > 0.05) were observed in metabolic activity as a function of any GF and/or CR supplementation at any timepoint (**Supplementary Figure S2**). IGF1 (at days 10 and 13) and TGF*β*3 (at days 7, 10 and 13) supplementation (without/with CR) significantly (*p* < 0.05) increased nuclei number (**Supplementary Figure S3**). PDGF*ββ* and GDF5 (at days 7, 10 and 13) supplementation without CR and in serial fashion to CR significantly (*p* < 0.05) increased cell nuclei number (**Supplementary Figure S3**).

### 3.2 Collagen synthesis and deposition analysis

**Fig. 1:**
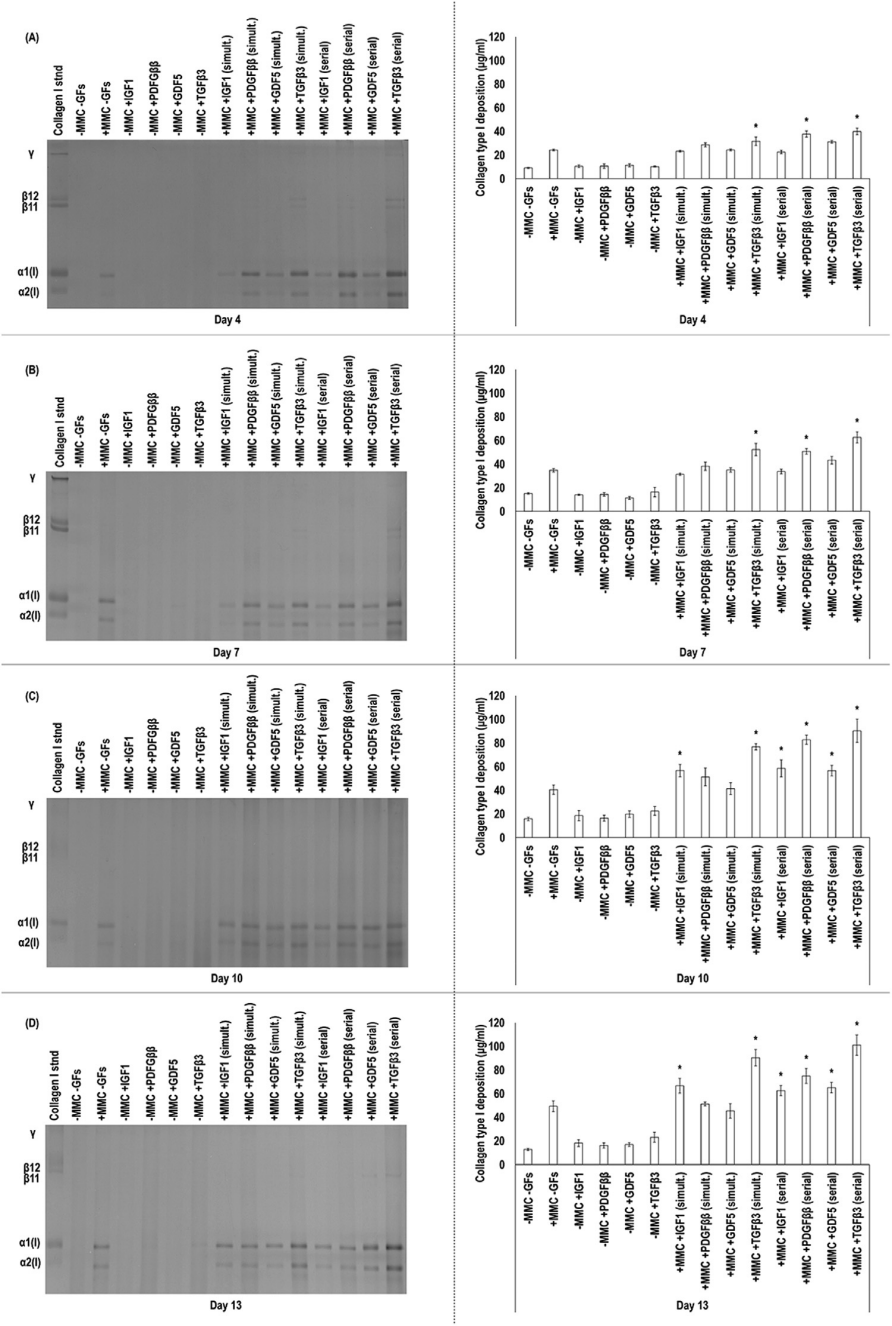
SDS-PAGE and densitometric analyses of cell layers of human tenocytes treated without (−)/with (+) MMC (carrageenan) and without (−)/with (+) (either in simultaneous or in serial fashion to MMC) IGF1, PDGF*ββ*, GDF5 and TGF*β*3 revealed that MMC supplementation, at all timepoints, induced significantly (*p* < 0.05) higher collagen type I deposition than the non-MMC groups (without or even with any GF). among the simultaneous GF supplementation to MMC, TGF*β*3 induced the highest (*p* < 0.05) collagen type I deposition in human tenocyte cultures at all time points. among the serial GF supplementation to MMC, TGF*β*3 induced the highest (*p* < 0.05) collagen type I deposition in human tenocyte cultures at all time points. Day 4 (**A**), Day 7 (**B**), Day 10 (**C**), Day 13 (**D**). * indicates significantly (*p* < 0.05) higher difference between the simultaneous and/or serial GF supplementation to MMC and MMC alone groups. Passage 3. *N* = 3.

SDS-PAGE and corresponding densitometric analyses (Fig. 1) showed that CR supplementation induced significantly (*p* < 0.05) higher collagen type I deposition in human tenocyte cultures than the non-crowded groups (without or with IGF1, PDGF*ββ*, GDF5 or TGF*β*3) at all timepoints.

Addition of TGF*β*3 alone at days 10 and 13 and IGF1 alone at day 13 induced significantly (*p* > 0.05) higher collagen type I deposition than the non-crowded groups (Fig. 1), whilst treatment with PDGF*ββ* or GDF5 alone did not enhance collagen type I deposition at any timepoint.

IGF1 and CR (in either simultaneous or serial fashion) induced significantly (*p* < 0.05) higher collagen type I deposition than IGF1 alone and no significant (*p* > 0.05) difference was observed between simultaneous and serial IGF1 to CR supplementation at any timepoint (Fig. 1). In comparison to CR alone, IGF1 and CR (in either simultaneous or serial fashion) induced significantly (*p* < 0.05) higher collagen type I deposition at days 10 and 13 (Fig. 1).

PDGF*ββ* and CR (in either simultaneous or serial fashion) induced significantly (*p* < 0.05) higher collagen type I deposition than PDGF*ββ* alone (Fig. 1). PDGF*ββ* in serial fashion to CR induced significantly (*p* < 0.05) higher collagen type I deposition than PDGF*ββ* in simultaneous fashion to CR at days 7, 10 and 13 (Fig. 1). In comparison to CR alone, only PDGF*ββ* in serial fashion to CR induced significantly (*p* < 0.05) higher collagen type I deposition at days 7, 10 and 13 (Fig. 1).

GDF5 and CR (in either simultaneous or serial fashion) induced significantly (*p* < 0.05) higher collagen type I deposition than GDF5 alone (Fig. 1). GDF5 in serial fashion to CR induced significantly (*p* < 0.05) higher collagen type I deposition than GDF5 in simultaneous fashion to CR at days 10 and 13 (Fig. 1). In comparison to CR alone, only GDF5 in serial fashion to CR induced significantly (*p* < 0.05) higher collagen type I deposition at days 10 and 13 (Fig. 1).

TGF*β*3 and CR (in either simultaneous or serial fashion) induced significantly (*p* < 0.05) higher collagen type I deposition than TGF*β*3 alone and no significant (*p* > 0.05) difference was observed between simultaneous and serial TGF*β*3 to CR supplementation at any timepoint (Fig. 1). In comparison to CR alone, TGF*β*3 and CR (in either simultaneous or serial fashion) induced significantly (*p* < 0.05) higher collagen type I deposition at days 7, 10 and 13 (Fig. 1).

**Fig. 2:**
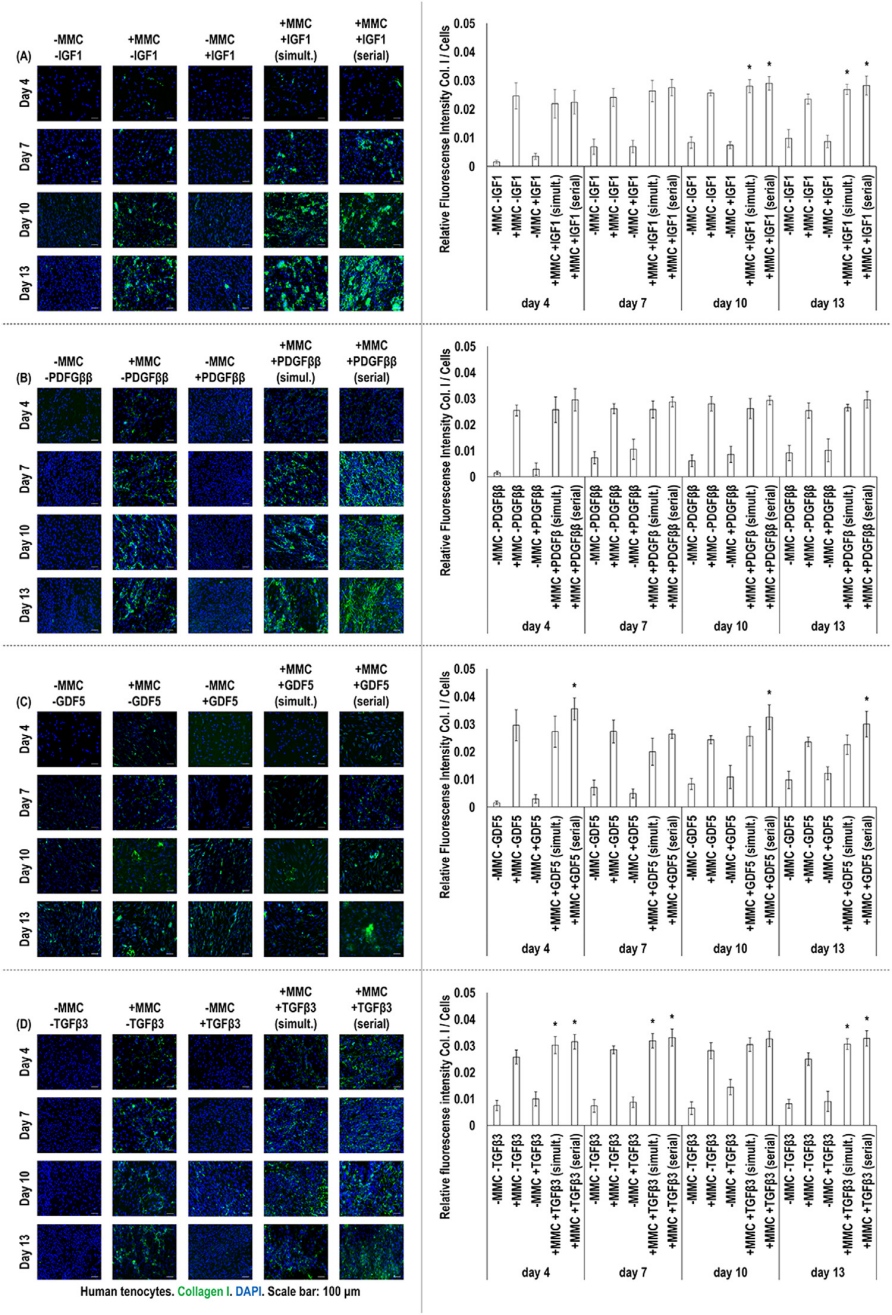
Immunocytochemistry and relative fluorescence intensity analyses of collagen type I of cell layers of human tenocytes treated without (−)/with (+) MMC (carrageenan) and without (−)/with (+) (either in simultaneous or in serial fashion to MMC) IGF1, PDGF*ββ*, GDF5 and TGF*β*3 revealed that MMC supplementation, at all timepoints, induced significantly (*p* < 0.05) higher collagen type I deposition than the non-MMC groups (without or even with any GF). At day 10 and 13, IGF1 in both simultaneous and serial to MMC supplementation induced significantly higher (*p* < 0.05) collagen type I deposition than the MMC alone group (**A**). No significant (*p* > 0.05) difference was detected in collagen type I deposition between MMC alone and PDGF*ββ* supplementation in either simultaneous or serial fashion to MMC (**B**). At days 4, 10 and 13, GDF5 supplementation in serial fashion to MMC induced significantly (*p* < 0.05) higher collagen type I deposition than the MMC alone group (**C**). At day 4, 7 and 13, TGF*β*3 supplementation in simultaneous and serial fashion to MMC induced significantly higher (*p* < 0.05) collagen type I deposition than the MMC alone group (**D**). * indicates significantly (at *p* < 0.05) higher difference between the simultaneous and/or serial GF supplementation to MMC and MMC alone groups. Collagen type I: Green, DAPI: Blue. Scale bars: 100 μm. Passage 3. *N* = 3.

**Fig. 3:**
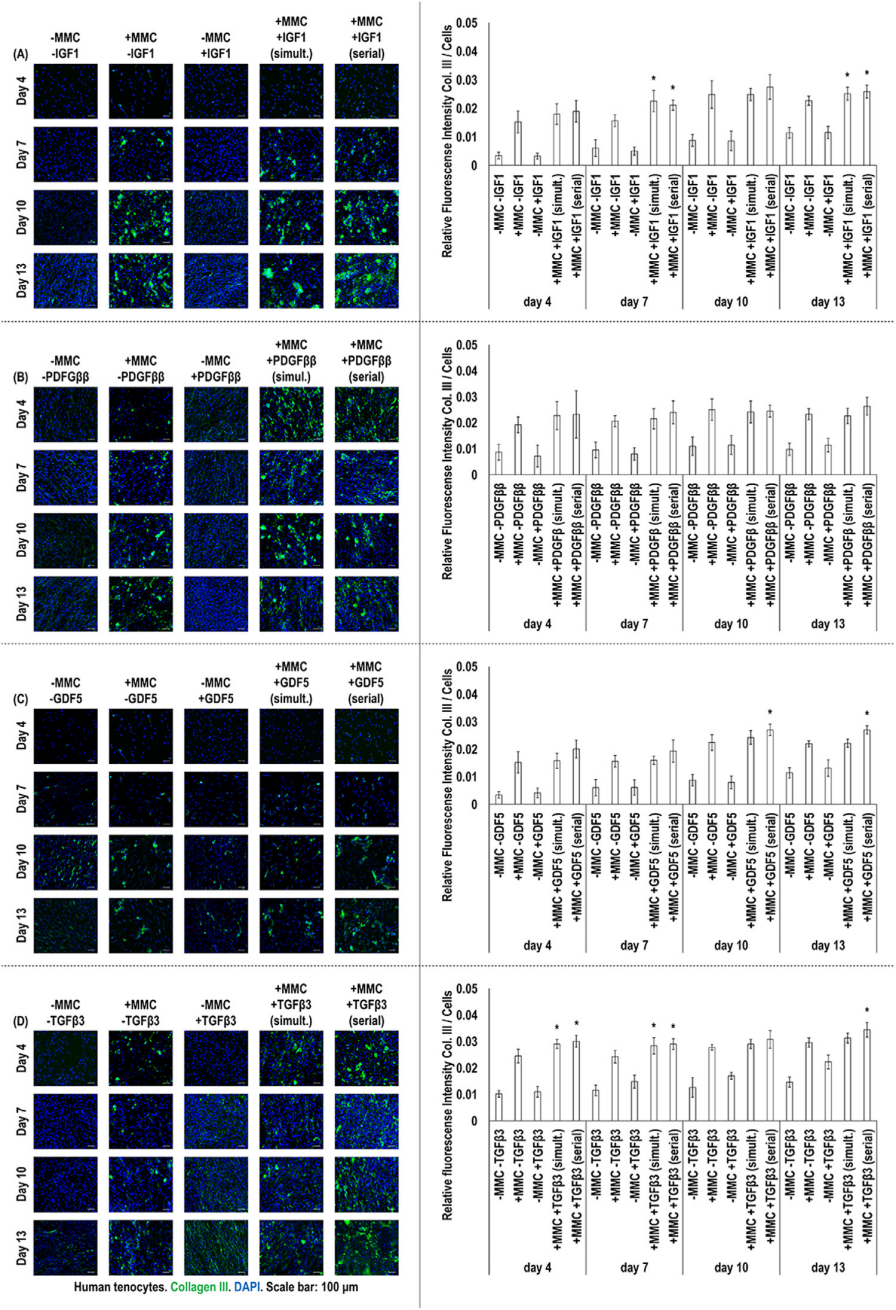
Immunocytochemistry and relative fluorescence intensity analyses of collagen type III of cell layers of human tenocytes treated without (−)/with (+) MMC (carrageenan) and without (−)/with (+) (either in simultaneous or in serial fashion to MMC) IGF1, PDGF*ββ*, GDF5 and TGF*β*3 revealed that MMC supplementation, at all timepoints, induced significantly (*p* < 0.05) higher collagen type III deposition than the non-MMC groups (without or even with any GF). At day 7 and, IGF1 supplementation in simultaneous and serial fashion to MMC induced significantly higher (*p* < 0.05) collagen type III deposition than the MMC alone group (**A**). No significant (*p* > 0.05) difference was detected in collagen type III deposition between MMC alone and PDGF*ββ* supplementation in either simultaneous or serial fashion to MMC (**B**). At day 10 and 13, GDF5 supplementation in serial fashion to MMC induced significantly (*p* < 0.05) higher collagen type III deposition than the MMC alone group (**C**). At day 4 and 7, TGF*β*3 supplementation either simultaneously or in serial fashion to MMC and at day 13 only in serial fashion to MMC induced significantly (*p* < 0.05) higher collagen type III deposition than the MMC alone group (**D**). * indicates significantly (at *p* < 0.05) higher difference between the simultaneous and/or serial GF supplementation to MMC and MMC alone groups. Collagen type III: Green. DAPI: Blue. Scale bars: 100 μm. Passage 3. *N* = 3.

Immunocytochemistry and corresponding fluorescent intensity analyses (collagen type I: Fig. 2, collagen type III: Fig. 3) revealed that CR supplementation induced significantly (*p* < 0.05) higher collagen type I and collagen type III deposition than the non-crowded groups (without or with IGF1, PDGF*ββ*, GDF5 or TGF*β*3) at all timepoints. No significant (*p* > 0.05) difference was detected in collagen type I (Fig. 2) and collagen type III (Fig. 3) deposition between simultaneous and serial IGF1, PDGF*ββ*, GDF5 or TGF*β*3 to CR supplementation at any timepoint. In comparison to CR alone, IGF1 in both simultaneous and serial supplementation to CR induced significantly higher (*p* < 0.05) collagen type I (at days 10 and 13, Fig. 2) and collagen type III (at days 7 and 13, Fig. 3) deposition. No significant (*p* > 0.05) difference was detected in collagen type I (Fig. 2) and collagen type III (Fig. 3) deposition between CR and PDGF*ββ* to CR simultaneous or serial supplementation at any timepoint. In comparison to CR alone, GDF5 in serial supplementation to CR induced significantly higher (*p* < 0.05) collagen type I (at days 4, 10 and 13, Fig. 2) and collagen type III (at days 10 and 13, Fig. 3) deposition. In comparison to CR alone, TGF*β*3 in both simultaneous and serial supplementation to CR induced significantly higher (*p* < 0.05) collagen type I (at days 4, 7 and 13, Fig. 2) and collagen type III [at days 4, 7 and 13 (only the serial), Fig. 3] deposition.

### 3.3 Gene expression analysis

**Fig. 4:**
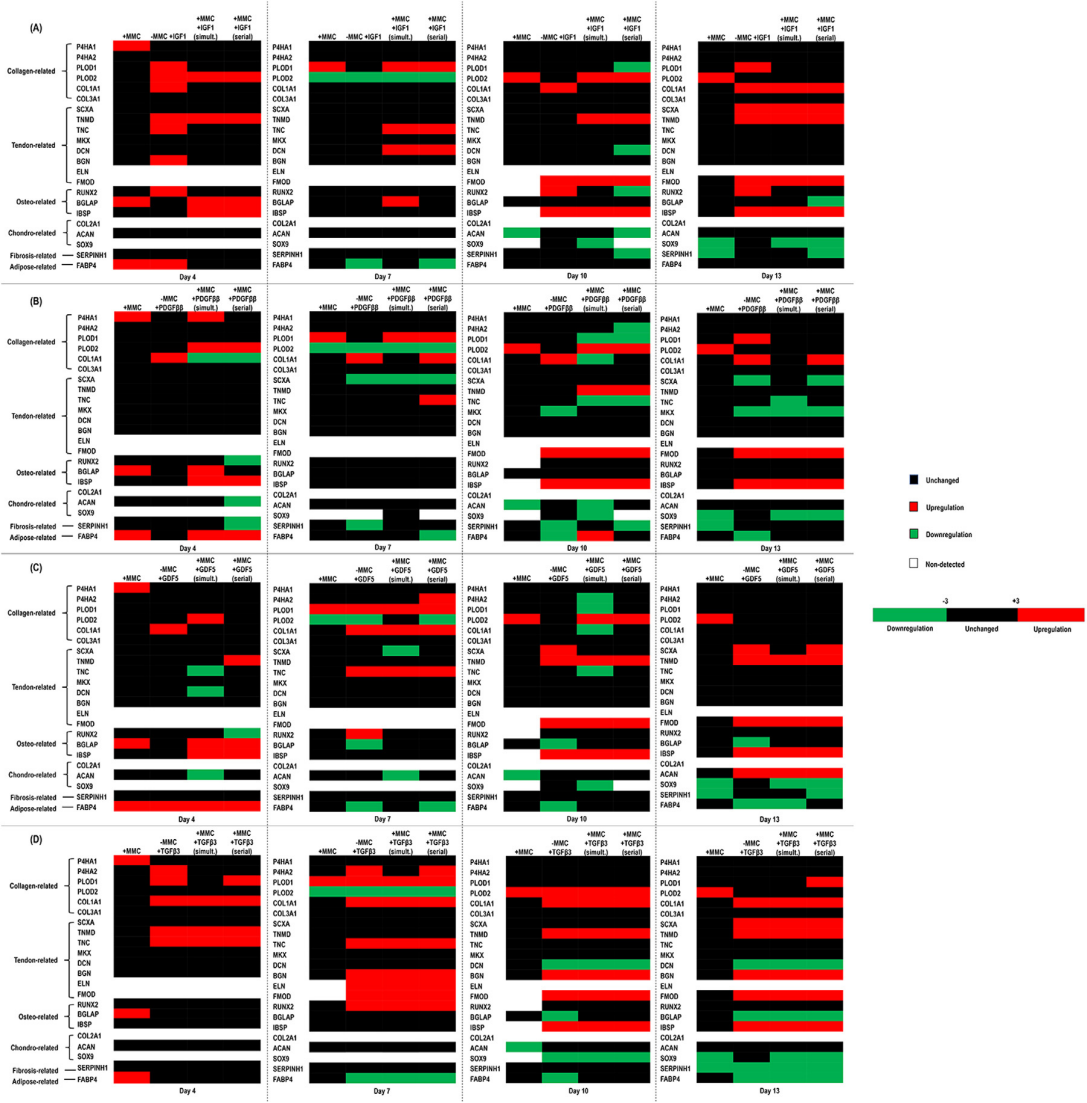
Hierarchal clustering of the fold change (threshold of 3) in gene expression of human tenocytes cultured without (−)/with (+) MMC (carrageenan) and without (−)/with (+) (either in simultaneous or in serial fashion to MMC) IGF1, PDGF*ββ*, GDF5 and TGF*β*3 revealed that at all timepoints TGF*β*3 alone and TGF*β*3 in either simultaneous or serial supplementation to CR upregulated the most and downregulated the least collagen- and tendon- related genes and upregulated the least and downregulated the most osteo-, chondro-, fibrosis- and adipose- related trans-differentiation genes in comparison to the other GFs without CR and with CR (either in simultaneous or serial fashion to the GFs). IGF1 (**A**), PDGF*ββ* (**B**), GDF5 (**C**), TGF*β*3 (**D**). The heatmap was generated by a log transformation of the real-time PCR data presented as ΔCΤ = (CΤ miRNA – CΤ GAPDH) compared to without CR and without GF at each timepoint. Passage 3. Data derived by pooling six wells per sample (*N* = 2).

Hierarchal clustering of the fold change (threshold of 3) in gene expression of human tenocytes cultured with CR in comparison to cells cultured without CR revealed that most genes were unchanged, except of P4HA1, BGLAP and FABP4 at day 4, PLOD1 at day 7, PLOD2 at day 10 and PLOD2 at day 13, which were upregulated and PLOD2 at day 7, ACAN at day 10 and SOX9 and SERPINH1 at day 13, which were downregulated (Fig. 4).

Hierarchal clustering of the fold change (threshold of 3) in gene expression of human tenocytes cultured with IGF1 without CR and with CR (either in simultaneous or serial fashion to IFG1) revealed that at day 4 IGF1 alone upregulated 3 collagen-related (PLOD1, PLOD2, COL1A1), 3 tendon-related (TNMD, TNC, BGN), 1 osteo-related (RUNX2) and 1 adipose-related (FABP4) genes; at day 7 IGF1 in serial fashion to CR upregulated 1 collagen-related (PLOD1) and 2 tendon-related (TNC, DCN) genes and downregulated 1 adipose-related (FABP4) gene; at day 10 IGF1 in serial fashion to CR upregulated 1 collagen-related (PLOD2), 2 tendon-related (TNMD, FMOD) and 1 osteo-related (IBSP) and downregulated 1 collagen-related (PLOD1), 1 tendon-related (DCN), 1 osteo-related (RUNX2), 1 chondro-related (ACAN) and 1 fibrosis-related (SERPINH1) genes; and at day 13 IGF1 in serial fashion to CR upregulated 1 collagen-related (COL1A1), 3 tendon-related (SCXA, TNMD, FMOD) and 1 osteo-related (IBSP) and downregulated 1 osteo-related (BGLAP), 1 chondro-related (SOX9) and 1 fibrosis-related (SERPINH1) genes (Fig. 4**A**).

Hierarchal clustering of the fold change (threshold of 3) in gene expression of human tenocytes cultured with PDGF*ββ* without CR and with CR (either in simultaneous or serial fashion to PDGF*ββ*) revealed that at day 4 PDGF*ββ* alone upregulated 1 collagen-related (COL1A1) gene; at day 7 PDGF*ββ* in serial fashion to CR upregulated 2 collagen-related (PLOD1, COL1A1) and 1 tendon-related (TNC) genes and downregulated 1 collagen-related (PLOD2), 1 tendon related (SCXA) and 1 adipose-related (FABP4) genes; at day 10 PDGF*ββ* in simultaneous fashion to CR upregulated 1 collagen-related (PLOD2), 2 tendon-related (TNMD, FMOD), 1 osteo-related (IBSP) and 1 adipose-related (FABP4) genes and downregulated 2 collagen-related (PLOD1, COL1A1), 1 tendon-related (TNC) and 2 chondro-related (ACAN, SOX9) genes; and at day 13 PDGF*ββ* alone upregulated 2 collagen-related (PLOD1, COL1A1), 1 tendon-related (FMOD) and 1 osteo-related (IBSP) genes and downregulated 2 tendon-related (SCXA, MKX) and 1 adipose-related (FABP4) genes (Fig. 4**B**).

Hierarchal clustering of the fold change (threshold of 3) in gene expression of human tenocytes cultured with GDF5 without CR and with CR (either in simultaneous or serial fashion to GDF5) revealed that at day 4 GDF5 alone upregulated 1 collagen-related (COL1A1) and 1 adipose-related (FABP4) genes; at day 7 GDF5 in serial fashion to CR upregulated 3 collagen-related (P4HA2, PLOD1, COL1A1) and 1 tendon-related (TNC) genes and downregulated 1 collagen-related (PLOD2) and 1 adipose-related (FABP4) genes; at day 10 GDF5 alone upregulated 3 tendon-related (SCXA, TNMD, FMOD) and 1 osteo-related (IBSP) genes and downregulated 1 osteo-related (BGLAP) and 1 adipose-related (FABP4) genes; and at day 13 GDF5 alone upregulated 3 tendon-related (SCXA, TNMD, FMOD), 1 osteo-related (IBSP) and 1 chondro-related (ACAN) genes and downregulated 1 osteo-related (BGLAP) and 1 adipose-related (FABP4) genes and GDF5 in serial fashion to CR upregulated 3-tendon-related (SCXA, TNMD, FMOD), 1 osteo-related (IBSP) and 1 chondro-related (ACAN) genes and downregulated 1 chondro-related (SOX9) and 1 fibrosis-related (SERPINH1) genes (Fig. 4**C**).

Hierarchal clustering of the fold change (threshold of 3) in gene expression of human tenocytes cultured with TGF*β*3 without CR and with CR (either in simultaneous or serial fashion to TGF*β*3) revealed that at day 4 TGF*β*3 alone upregulated 3 collagen-related (P4HA2, PLOD1, COL1A1) and 2 tendon-related (TNMD, TNC) genes; at day 7 TGF*β*3 alone and TGF*β*3 in serial fashion to CR upregulated 3 collagen-related (P4HA2, PLOD1, COL1A1), 4 tendon-related (TNC, BGN, ELN, FMOD) and 1 osteo-related (RUNX2) genes and downregulated 1 collagen-related (PLOD2) and 1 adipose-related (FABP4) genes; at day 10 TGF*β*3 alone upregulated 2 collagen-related (PLOD2, COL1A1), 3 tendon-related (TNMD, BGN, FMOD) and 1 osteo-related (IBSP) genes and downregulated 1 tendon-related (DCN), 1 osteo-related (BGLAP), 1 chondro-related (SOX9) and 1 adipose-related (FABP4) genes; and at day 13 TGF*β*3 in serial fashion to CR upregulated 2 collagen-related (PLOD1, COL1A1), 4 tendon-related (SCXA, TNMD, BGN, FMOD) and 1 osteo-related (IBSP) genes and downregulated 1 tendon-related (DCN), 1 osteo-related (BGLAP), 1 chondro-related (SOX9), 1 fibrosis-related (SERPINH1) and 1 adipose-related (FABP4) genes (Fig. 4**D**).

## 4 Discussion

Although tenocytes are the cell population of choice in tendon tissue engineering, their clinical potential is hampered by their susceptibility to phenotypic drift and loss of functionality with passaging. Multifactorial approaches are gaining popularity to either maintain tenocyte phenotype [65] or to direct stem cells towards tenogenic lineage [66]. among the various *in vitro* microenvironment modulators, GF supplementation is most likely the most potent method to maintain phenotype, whilst MMC is the certainly the most potent method to enhance ECM deposition. Thus, herein, we evaluated the combining effect of GF and MMC supplementation in human tenocyte culture. We chose GFs relevant to tendon morphogenesis, development and healing. For example, IGF1 and PDGF*ββ* have multiple roles during the inflammatory and proliferative stages of tendon healing after injury [67], [68], [69]; GDF5, a member of the bone morphogenetic protein (BMP) family, is a crucial regulator of tendon development [70,71]; and TGF*β*3 acts as a crucial mediator of tendon morphogenesis [72], [73], [74]. CR was selected as MMC agent, as due to its inherent polydispersity most effectively excludes volume and thus induces the highest ECM deposition in the shortest period of time [51].

Starting with simultaneous versus serial GF supplementation to CR, notable differences were observed throughout the study. For example, PDGF*ββ* and GDF5 in serial fashion to CR induced higher DNA content and proliferation and all GFs in serial fashion to CR induced higher collagen deposition and resulted in some differences in gene expression. These observations are in agreement with previous reports that have shown various isoforms of CR to selectively bind and interact with GFs (e.g. heparin-binding haematopoietic growth factors [75]; basic fibroblast growth factor, TGF*β*1, TGF*α* and PDGF*ββ*, but not IGF1 [76,77], although other studies have shown CR to inhibit insulin signalling [78,79]). Considering that GDF5 belongs to TGF*β* superfamily, which binds to heparin/heparin sulphate with high affinity [80], it is plausible that CR also acts as a GDF5 antagonist. It is also worth noting that although TGF*β*1/2 is able to bind to heparin and heparin sulphate, TGF*β*3 does not interact with these [81], suggesting, in our case, that CR did not inhibit the effect of TGF*β*3. Overall, our data are in agreement with previous observation showing the ability of polysaccharides, in particular sulphated polysaccharides, to modulate GF activity (e.g. FGF2 [82], [83], [84], [85], IGF1 [86,87], TGF family [88], [89], [90], PDGF*ββ* [91, 92], GDF5 [93]) and subsequently influence cell response. Indeed, it has been suggested that due to the structural similarity of sulphated polysaccharides with heparin, sulphated polysaccharides interact with the heparin binding domain of the GFs rather than their receptors on the cells [77], resulting in their stabilisation, potentiation of their activity and even in their controlled delivery [94].

Another notable result is that CR alone induced significantly higher collagen deposition not only over the non-crowded groups, but also over the non-crowded groups supplemented with the various GFs. The effectiveness of CR to enhance and accelerate ECM deposition has been shown in permanently differentiated [51,54,55] and stem [58,62,95] cell populations. In general, growth factors are used as phenotype maintenance/induction modulators (for indicative examples of GF supplementation in tendon space please see these references [43,96,97] and others cited herein), but also as a means to enhance ECM deposition (e.g. TGF [98,99], PDGF [100], GDF [101], IGF1 [102]). Clearly, data presented herein indicate that if enhanced and accelerated ECM deposition is the aim of a study, MMC should be utilised, as opposed to expensive and rather ineffective biological supplements. With respect to the complimentary action of MMC to GF supplementation, we attribute this to the excluded volume effect that MMC induced and resulted in reduced diffusion of the GFs in the culture media and enhanced their biological potential. Indeed, MMC has been shown to reduce diffusion rates [103], [104], [105] by obstructing molecular motion [106], [107], [108], [109] in physicochemical properties (e.g. concertation, viscosity, size, shape, charge) dependent manner.

In all cases, metabolic activity remained constant, whilst DNA content and cell proliferation were increased as a function of time in culture and optimal (simultaneous or serial) GF supplementation. Collagen type I deposition (via SDS-PAGE analysis, normalised to the standard) was also increased as a function of time in culture for the CR and GF supplemented groups. Although collagen type I and collagen type III deposition (via immunocytochemistry analysis, normalised to the cell number) did not change as a function of time in culture, they were increased with CR alone at all timepoints and were increased further with optimal (simultaneous or serial) GF supplementation by day 13 for all CR and GF treatments. These data are in agreement with previous studies, where IGF1 [39,40,110], PDGF*ββ* [31,41,111,112], GDF5 [32,113] and TGF*β*3 [114,115] supplementation have been shown to increase DNA content, proliferation and/or ECM synthesis *in vitro* and *in vivo*. Of significant importance is the observation that among the groups, TGF*β*3 in simultaneous or serial fashion to CR appeared to induce collagen deposition. It is well known that TGF*β* family of growth factors stimulates the expression of collagen and fibronectin and their relative incorporation into the matrix of various types of fibroblasts [116], mainly through the regulation of TGF-*β*/Smad signalling pathway [117].

With respect to gene analysis, CR alone appeared to upregulate at days 10 and 13 collagen-related genes (PLOD2) and to downregulate cartilage (ACAN at day 10 and SOX9 at day 13) and fibrosis (SERPINH1) trans-differentiation genes. The hydroxylation of lysyl residues is a critical step in collagens biosynthesis and occurs at the Y position of the repeating Gly-X-Y sequence. PLOD1, PLOD2 and PLOD3 catalyse the hydroxylation of lysine to hydroxylysine, which is critical for the formation and stabilisation of covalent crosslinks, mechanical resilience and collagen fibre alignment, especially in hypoxic conditions [118], [119], [120]. As it has been shown before [55], the addition of CR did not appear to promote fibrosis, but rather reparative processes. Further, although previous studies have shown sulphated polysaccharides to enhance chondrogenesis (via alkaline phosphatase inhibition) and osteogenesis (via BMP2 and RUNX2 enhancement) in various cell populations [121], [122], [123], [124], [125], [126], this was not observed herein. Overall, these data clearly advocate the use of CR as MMC in human tenocyte culture.

At all timepoints, gene expression analysis made apparent that TGF*β*3 (alone or in combination with CR) more effectively than the other GFs maintained tenocyte phenotype. Further, at day 13, TGF*β*3 in simultaneous or serial fashion to CR appeared to most effectively maintain tenocyte phenotype, as judged by upregulation of collagen- [PLOD1 (was unchanged for the simultaneous treatment), COL1A1] and tendon- (SCXA, TNMD, BGN, FMOD) related genes, downregulation of osteo- (BGLAP), chondro- (SOX9), fibrosis- (SERPINH1) and adipose- (FABP4) related trans-differentiation genes, upregulation of the least number of osteo-related (IBSP) trans-differentiation genes and downregulation of the least number of tendon-related (DCN) genes. Similar to our data, the beneficial effects of TGF*β*3 in maintenance of tendon cell fate has been well-established in the literature [43]. It is also worth noting that TGF*β*3 supplementation to tenocyte cultures has been shown to downregulate Smad3 and to upregulate Smad7, minimising that way extrinsic scarring [127], further corroborating our findings of reduced expression of SERPINH1. Considering the beneficial effects of PLOD in collagen synthesis, stabilisation and alignment [128], especially in low oxygen conditions [129,130], like tendons, we believe that TGF*β*3 supplementation in serial fashion to CR may be the most appropriate mode of administration.

The observed upregulation of IBSP and downregulation of DCN are associated with the TGF*β*3 supplementation. Indeed, fluid dynamic shear stress has been shown to increase various GF secretion, including TGF, which resulted in upregulation of osteogenic markers, including IBSP in human alveolar bone-derived mesenchymal stem cells [131]. Similarly, previous studies have demonstrated the inhibitory interaction between decorin and TGF*β*1 [132,133]. Obviously, upregulation of IBSP and downregulation of DCN are of concern, but further media refinement may alleviate these downfalls. To substantiate this, a previous study, for example, has shown TGF*β*3 and IGF1 (the second most effective GF in this study) supplementation to increase DCN expression in rat nucleus pulposus-derived mesenchymal stem cell cultures [134]. An alternative approach may be a two-stage process, comprising of a first TGF*β*3 step, followed by a TGF*β*3-free step as has been previously suggested [43]. GF cocktails may also be the way forward, considering that cells are exposed simultaneously to numerous biological stimuli *in vivo* and numerous studies have advocated the beneficial effects of GF cocktails in tenocyte phenotype maintenance [135,136]. In either case, considering that data presented herein demonstrate the synergistic effect of GF and MMC supplementation, we feel that MMC should be appropriately incorporated into the experimental design.

We appreciate that these data may be in contradiction to previous studies were the use of IGF1, PDGF*ββ* or GDF5 were advocated. However, none of these studies had assessed trans-differentiation genes (8 trans-differentiation genes were assessed herein) and the conclusions were based only on 4–15 collagen- and tendon- related genes [31,32,97,113] (we also assessed herein 14 collagen- and tendon- related genes). Considering the well-established plasticity of tenocytes, as we have suggested before [19,20], to safely conclude on the effectiveness of an approach to maintain/induce tenogenic phenotype, trans-differentiation markers should also be assessed.

## 5 Conclusions

In the context of tendon tissue engineering, clinical translation and commercialisation of tenocyte-based therapies is hampered by their prolonged culture time required to develop a three-dimensional implantable device, which is associated with tenocyte loss of function. Herein, we demonstrated that growth factor (TGF*β*3) supplementation in serial fashion to macromolecular crowding (carrageenan), synergistically contributed to maintaining physiological tenocyte function. Our data further corroborate the notion towards multifactorial tissue engineering strategies for the development of functional cell-assembled tissue-like equivalents.

## Declaration of Competing Interest

The authors declare no conflict of interest.

## Appendix Supplementary materials

Image, application 1
