# Supplementary material for: Growth factor and macromolecular crowding supplementation in human tenocyte culture
Source: Biomater Biosyst. 2021 Jan 30;1:100009. doi: 10.1016/j.bbiosy.2021.100009 (PMC9934496; doi:10.1016/j.bbiosy.2021.100009)
Supplement: Supplementary file 1 [file mmc1.docx]

**Supplementary Information**

# Title

Growth factor and macromolecular crowding supplementation in human tenocyte culture

# Authors

Dimitrios Tsiapalis (1, 2), Stephen Kearns (3), Jack L. Kelly (4), Dimitrios I. Zeugolis^[[1]](#footnote-1)^ (1, 2, 5)

# Affiliations

1. Regenerative, Modular & Developmental Engineering Laboratory (REMODEL), Biomedical Sciences Building, National University of Ireland Galway (NUI Galway), Galway, Ireland

2. Science Foundation Ireland (SFI) Centre for Research in Medical Devices (CÚRAM), Biomedical Sciences Building, National University of Ireland Galway (NUI Galway), Galway, Ireland

3. Merlin Park University Hospital, Galway, Ireland

4. Galway University Hospital, Galway, Ireland

5. Regenerative, Modular & Developmental Engineering Laboratory (REMODEL), Faculty of Biomedical Sciences, Università della Svizzera Italiana (USI), Lugano, Switzerland

**Supplementary Table S1:** List of genes and sequence of their respective primers.

| **Gene Name** | **Gene Symbol** | **Forward Sequence** | **Reverse Sequence** |
| --- | --- | --- | --- |
| Prolyl 4-hydroxylase subunit alpha 1 | P4HA1 | TGAAATCGTCAAAGACCTAGCA | TGTTATTGGGTTTGAAATGGTG |
| Prolyl 4-hydroxylase subunit alpha 2 | P4HA2 | AAACTGGTGAAGCGGCTAAA | GAGAGGTTGGCGATAAAACCT |
| Procollagen-lysine,2-oxoglutarate 5-dioxygenase 1 | PLOD1 | GCTGCCGTATCTTCCAGAAC | TTTCAAACTTGAGCACGACCT |
| Procollagen-lysine,2-oxoglutarate 5-dioxygenase 2 | PLOD2 | AAGGACTTTAAAAATTTTGATTGAACA | GACTCAATGCTCCCCAGAAAT |
| Collagen type I alpha 1 | COL1A1 | AGGTGAAGCAGGCAAACCT | CTCGCCAGGGAAACCTCT |
| Collagen type III alpha 1 | COL3A1 | ACTGGAGCACGGGGTCTT | TCCTGGTTTCCCACTTTCAC |
| Scleraxis homolog A | SCXA | CCCAAACAGATCTGCACCTT | TCTTTCTGTCGCGGTCCTT |
| Tenomodulin | TNMD | TCCTCTGGCATCTGTTAGCC | TCCTTGCTTTGAGAGGACTGA |
| Tenascin C | TNC | CCTTGCTGTAGAGGTCGTCA | CCAACCTCAGACACGGCTA |
| Mohawk homeobox | MKX | GGATCCAATAAGGGTGAAAGC | TAAGGCCATAGCTGCGTTG |
| Decorin | DCN | CCAATATCACCAGCATTCCTC | CTGCTGATTTTGTTGCCATC |
| Biglycan | BGN | CTACAGCGCCATGTGTCCT | TCTTTGGGCACAGACTTCAG |
| Elastin | ELN | CACTGGGGTATCCCATCAAG | GTGGTGTAGGGCAGTCCATAG |
| Fibromodulin | FMOD | TCCAAGGCAATAGGATCAATG | AGTTCACGACGTCCACCAC |
| Runt related transcription factor 2 | RUNX2 | TGCCACCTCTGACTTCTGC | AAAGGGCCCAGTTCTGAAG |
| Bone gamma-carboxyglutamate (gla) protein | BGLAP | CCAGCCCTATGGATGTGG | TTTTCAGATTCCTCTTCTGGAGTT |
| Integrin-binding sialoprotein | IBSP | AGAACCACTTCCCCACCTTT | TTCGTACTCCCCCTCGTATTC |
| Collagen type II alpha 1 | COL2A1 | CTGGTCCTCAAGGCAAAGTT | GAGGTCCAGGACGACCATC |
| Aggrecan | ACAN | GAACGACAGGACCATCGAA | AAAGTTGTCAGGCTGGTTGG |
| SRY (sex determining region Y)-box 9 | SOX9 | TACCCGCACTTGCACAAC | TCTCGCTCTCGTTCAGAAGTC |
| Serpin H1 precursor (heat shock protein 47) | SERPINH1 | ATGCAGAAGAAGGCTGTTGC | CTTGTCAATGGCCTCAGTCA |
| Fatty acid binding protein 4 | FABP4 | CCTTTAAAAATACTGAGATTTCCTTCA | AGGACACCCCCATCTAAGGT |
| **Housekeeping genes** | | | |
| Glyceraldehyde-3-phosphate dehydrogenase | GAPDH | AGCCACATCGCTCAGACAC | GCCCAATACGACCAAATCC |
| Actin beta | ACTB | TCCTCCCTGGAGAAGAGCTA | CGTGGATGCCACAGGACT |
| Ribosomal protein, large, P0 | RPLP0 | TCGACAATGGCAGCATCTAC | GCCAATCTGCAGACAGACAC |

**Supplementary Figure S1:** In all cases, DNA content was increased as a function of time in culture. At days 10 and 13, the IGF1 treatments (without / with MMC, carrageenan) exhibited significantly (*p* < 0.05) higher DNA content than the non-IGF1 treatments (**A**). At all timepoints, the PDGF*ββ* without MMC and the PDGF*ββ* in serial fashion to MMC treatments exhibited the highest (*p* < 0.05) DNA content (**B**). At days 7, 10 and 13, the GDF5 without MMC and the GDF5 in serial fashion to MMC treatments exhibited the highest (*p* < 0.05) DNA content (**C**). At all timepoints, the TGF*β*3 treatments (without / with MMC) exhibited significantly (*p* < 0.05) higher DNA content than the non-TGF*β*3 treatments (**D**). * indicates statistically significant (*p* < 0.05) difference as compared to without MMC and without GF treatments. Passage 3. N=3.


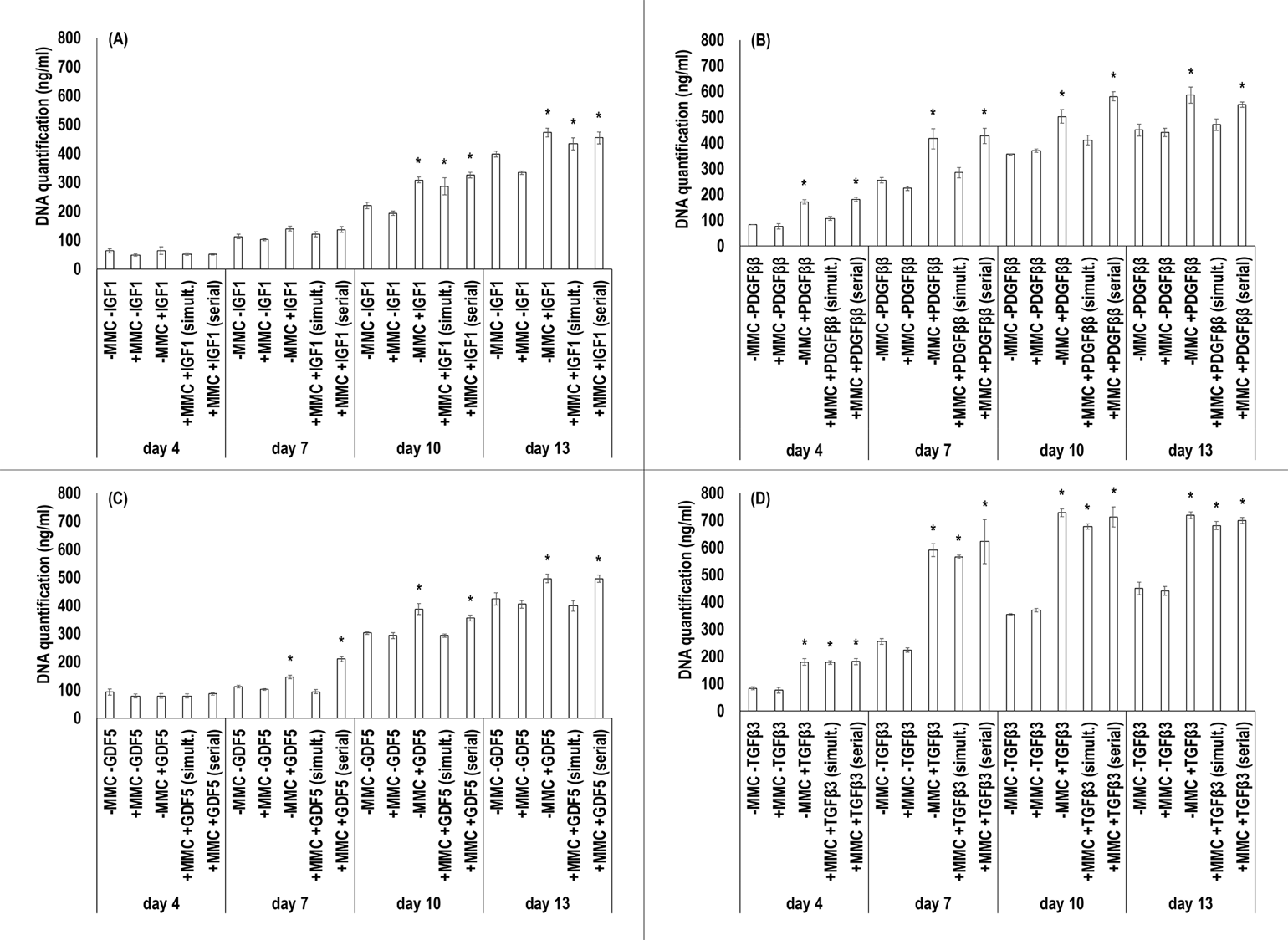


**Supplementary Figure S2:** No statistical differences (*p* > 0.05) in tenocyte metabolic activity were observed as a function of MMC (carrageenan) and/or GF (either in simultaneous or serial fashion to MMC) supplementation at any timepoint. IGF1 (**A**), PDGF*ββ* (**B**), GDF5 (**C**), TGF*β*3 (**D**). Passage 3. N=3.


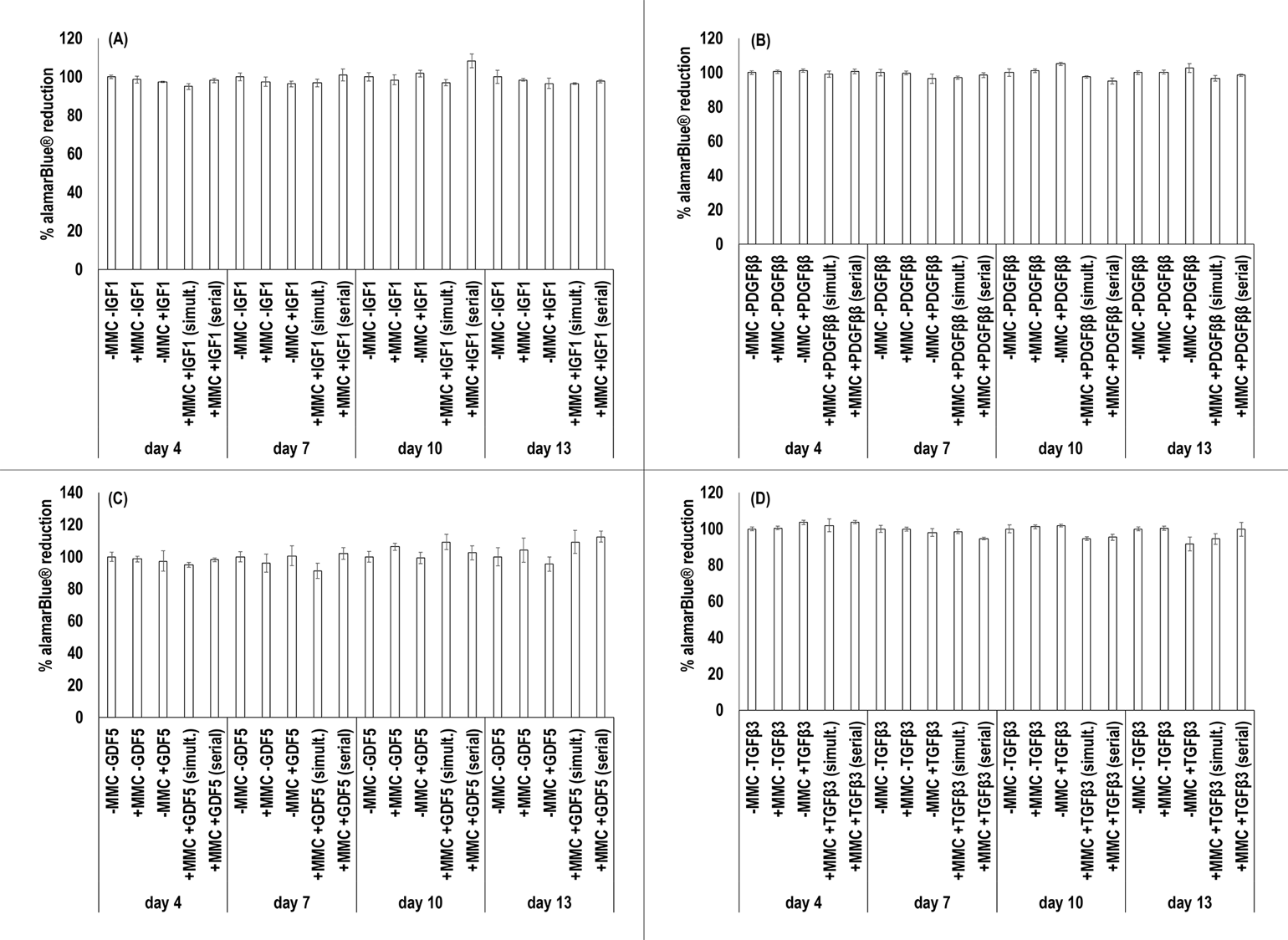


**Supplementary Figure S3:** In all cases, nuclei number was increased as a function of time in culture. At days 10 and 13, the IGF1 treatments (without / with MMC, carrageenan) exhibited significantly (*p* < 0.05) higher nuclei number than the non-IGF1 treatments (**A**). At days 7, 10 and 13, the PDGF*ββ* without MMC and the PDGF*ββ* supplemented in serial fashion to MMC treatments exhibited the highest (*p* < 0.05) nuclei number (**B**). At days 7, 10 and 13, the GDF5 without MMC and the GDF5 supplemented in serial fashion to MMC treatments exhibited the highest (*p* < 0.05) nuclei number (**C**). At all timepoints, the TGF*β*3 treatments (without / with MMC) exhibited significantly (*p* < 0.05) higher nuclei number than the non-TGF*β*3 treatments (**D**). * indicates statistically significant (*p* < 0.05) difference as compared to without MMC and without GF treatments. Passage 3. N=3.


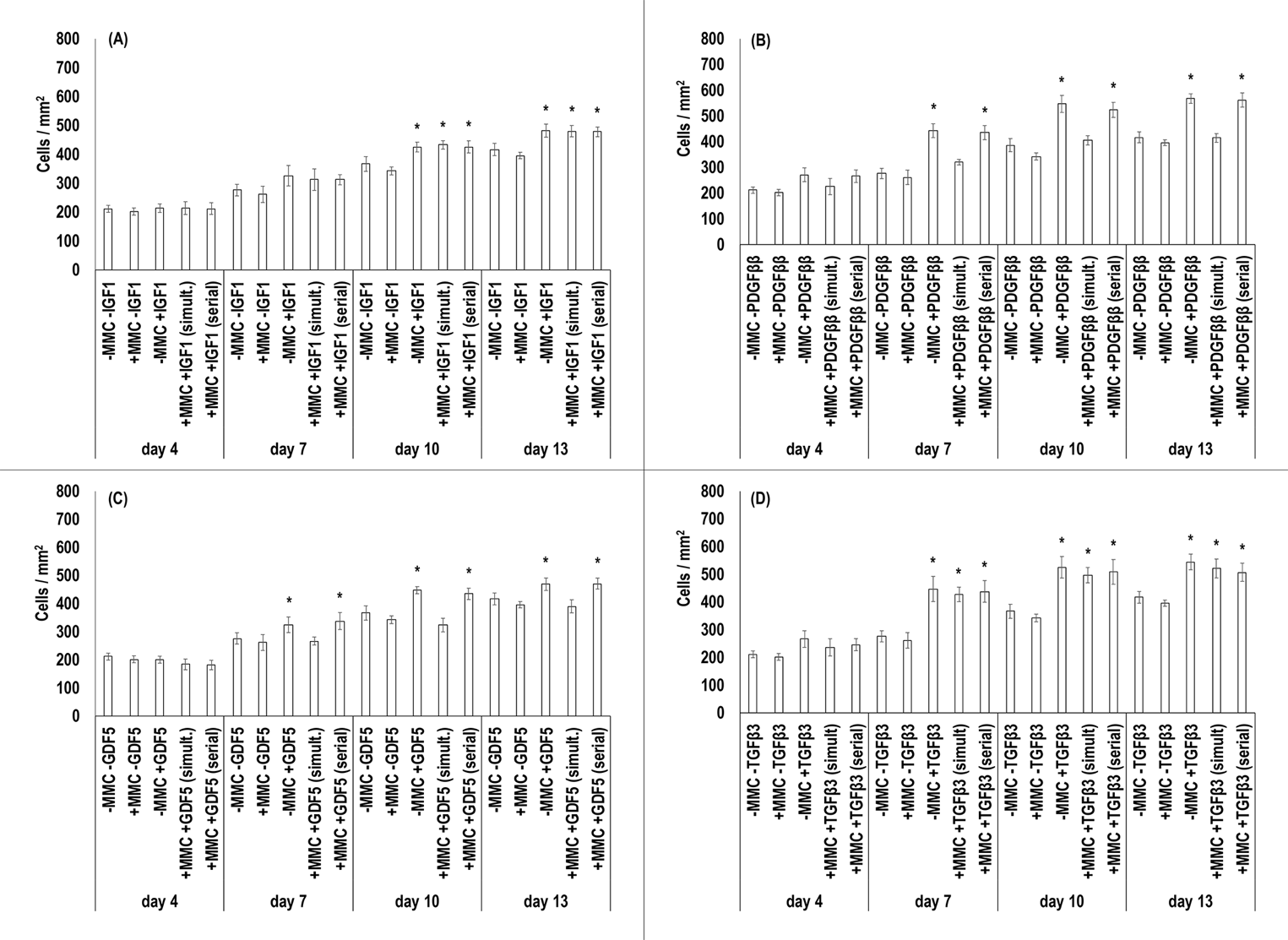


1. Corresponding Author: Dimitrios I. Zeugolis, REMODEL, NUI Galway & USI. Telephone: +41 58 666 40 00; Email: dimitrios.zeugolis@usi.ch [↑](#footnote-ref-1)
